# Anxiety Status and Coping Strategies in Association with Sociodemographic Factors, Dietary and Lifestyle Habits in Greece

**DOI:** 10.2174/1745017902117010152

**Published:** 2021-10-15

**Authors:** Maria Batsikoura, Sofia Zyga, Foteini Tzavella, Athanasios Sachlas, Andrea Paola Rojas Gil

**Affiliations:** 1 Department of Nursing, Laboratory of Biology and Biochemistry, Faculty of Health Sciences, University of Peloponnese, Tripoli, Greece; 2 Department of Nursing, Laboratory of Basic Nursing, Faculty of Health Sciences, University of Peloponnese, Tripoli, Greece; 3 Department of Nursing, Laboratory of Integrated Health Care, Faculty of Health Sciences, University of Peloponnese, Tripoli, Greece; 4 Department of Statistics and Insurance Science, University of Piraeus, Athens, Greece

**Keywords:** Anxiety, Dietary habits, Coping strategies, Stress, Behaviour, Lifestyle

## Abstract

**Aim:**

The aim of this study was to investigate the relationship between nutritional habits, lifestyle, anxiety, and coping strategies.

**Background:**

Anxiety is an underestimated and often undiagnosed subclinical disorder that burdens the general public of modern societies and increases illness suscentibility.

**Methods:**

The study group consisted of 693 individuals living in Peloponnese, Greece. A standardized questionnaire that consists of the dietary habits and lifestyle questionnaire, the trait Anxiety STAI-X-2 questionnaire and the brief-COPE questionnaire, was used. Principal components analysis identified the factors from the questionnaires, and stepwise multivariate regression analysis investigated their relationships.

**Results:**

Weekly consumption of fruits, tomatoes, salads and lettuce, together with Εmotional/Ιnstrumental support, Denial/Behavioural disengagement, substance use and self-blame, was the most important predictors of anxiety scores. Positive reframing/Humour and Acceptance/Planning are also associated with the Positive STAI factor and decreased anxiety scores.

**Conclusion:**

Healthy nutritional habits, comprised of consumption of salads and fruits, together with adaptive coping strategies, such as Positive reframing/Humour and Active problem solving, may provide the most profound improvement in the anxiety levels of a healthy population in Peloponnese, Greece.

## 1 INTRODUCTION

Anxiety is a very common but often misdiagnosed psychiatric problem in late adulthood, with subclinical anxiety symptoms being an even more frequent risk event [1]. The ever-increasing burden and prevalence of depression, estimated to reach more than 300 million people worldwide in coordination with its association with anxiety disorders [2], highlighting the importance of early diagnoses and appropriate treatment or even early prevention strategies. Anxiety disorders are reported to be the fourth cause of years lost due to disability [3], and the recent financial crisis in Europe has actively increased the risk factors related to anxiety in Greece, as well as in other European countries [4].

Anxiety disorders have been associated with impaired physical and role functioning; they adversely influence the course of chronic disease mainly through the adoption of unhealthy coping strategies and have been reported to increase illness susceptibility [5-7], indicating the importance of early intervention to relieve the burden of anxiety disorders in modern times.

Recent evidence shows that certain lifestyle strategies have beneficial effects on anxiety, but only a third of the people suffering usually seek any treatment [8]. Studies focusing on single food groups indicate that high fish, fruit, vegetable, and fibre consumption can significantly decrease depressive symptoms [9]. The benefits of a balanced diet and healthy lifestyle (exercising, adequate sleeping and moderate alcohol, junk food, and caffeine consumption) have been widely accepted to reduce and protect from the development of anxiety disorders and depression and their sustainability. In particular, practices such as a balanced nutrient load, appropriate hydration (in combination with limited alcohol and caffeine consumption), complex carbohydrate consumption, shown to control blood glucose levels, Magnesium (found in spinach, legumes, nuts, seeds, and whole grain), Zinc sources (like in cashews, liver, beef and egg yolks), Omega-3 fatty acids (found in fatty fish), Probiotics (from kefir and asparagus), rich vitamin B food (part of avocado and almonds) and Antioxidants (usually found in beans, berries, walnuts, broccoli artichoke, turmeric, and ginger), are all associated with lower anxiety and may help reduce anxiety symptoms, therefore becoming important dietary choices to implement in everyday life [9]. Recent nutritional research focusing on dietary patterns, reveals the importance of consuming protein and each association with a low prevalence of depression, possibly related to higher tryptophan levels, a serotonin precursor, in the brain [10, 11]. Furthermore, processed carbohydrates and sweets, may trigger high blood glucose and increase the physiological symptoms of stress, whereas low carb diets may decrease nutrient availability and initiate a cortisol response, resulting in anxiety. Therefore, minimally processed, complex carbohydrates that produce steady streams of glucose may minimize the effect of anxiety and should account for at least 20% of your daily caloric intake [12], and higher consumption of fibre, fish and Omega-3 fatty acids may be protective to anxiety and anxiety-related disorders [13]. Nutrients including tryptophan, vitamins B6, B12, A and C, folic acid, tyrosine, phenylalanine, histidine, glutamic acid and choline, are necessary for the production of neurotransmitters, primarily involved in mood, appetite and cognition, such as serotonin, dopamine and norepinephrine [14]. Healthy patterns that include Omega-3 fatty acids, vitamin B12, magnesium, zinc, vitamin D, C and E, iodine, selenium and protein may reduce anxiety and supplementation in certain cases, are highly recommended [15].

In addition, an increasing number of studies are providing important evidence that self-management also presents an important intervention strategy for decreasing the risk factors associated with anxiety and for treating mild anxiety [8].

The aim of this quantitative observational explorative research was to elucidate the characteristics of lifestyle, nutritional habits, medical history, and socioeconomic status, that strongly associate and affect anxiety scores and/or anxiety coping strategies, to extend and exploit new lifestyle and dietary intervention methods for mild anxiety in the general population.

## 2 MATERIALS AND METHODS

### 2.1 Participants and Sampling

The questionnaire was given to 701 adults (>18 years of age) from the Peloponnese region, Greece, between December 2015–December 2016. The response rate was 98.3%. The participants in the study were selected through a stratified sampling scheme according to the population size of two prefectures (Laconia and Messenia), firstly divided into building blocks and random sampling of those, secondly by systematic sampling of the households, and thirdly by random sampling in each household. Participants completed a set of the 3 self-reported questionnaires, with signed consent, after they were informed about the objectives of the study and were reassured about the confidentiality of their name, characteristics, and responses. The study was approved by the Ethics Committee of the School of Health Sciences, Department of Nursing, University of Peloponnese on 8/12/2017, while permissions were taken by the Hellenic Data Protection Authority on 25/9/2015, reference number ΓΝ/ΕΞ/4580-1/25-9-2015.

### 2.2 Measurements

A set of three questionnaires were given a hand by hand to the sample under study, including the dietary habits and lifestyle questionnaire [16], the State-Trait Anxiety Inventory (STAI-T)-X-2 form [17], and the brief Coping Orientation to Problems Experienced (COPE) inventory [18]. The dietary habits lifestyle questionnaire, is comprised of 75 closed-ended questions concerning the weekly dietary habits (weekly frequency and potion consumption) according to the following categories: a) junk food, b) meat, c) fruits and vegetables (categorised by their vitamin and mineral content), d) cereals, e) legumes, f) dairy products, g) beverages, and h) nuts, as well as their lifestyle including sleep, exercise, and smoking habits. All the above questionnaires have been previously translated and standardised in Greece [19-23].

The questionnaire also included variables assessing the socio-demographic characteristics of the participants. Exclusion criteria were inability to communicate in fluent Greek and psychiatric medication.

Each questionnaire was assessed in the first phase of the study in a pilot study of 100 participants for internal consistency and reliability, which was assessed by the Cronbach’s alpha internal consistency coefficient. Cronbach’s *α* was 0.812 for the dietary questionnaire, 0.710 for the STAI-X-2, and 0.806 for the brief-COPE. Therefore, the questionnaires were given to the full sample of 701 participants mentioned above.

For each questionnaire, Factor Analysis (FA) was employed, and the items of each questionnaire were reduced into certain factors. More analytically, the FA on the 19 variables of the dietary and lifestyle questionnaire (Table **S1**) led to six factors, indicating Fruits (cherries, watermelon, apricots, tangerines, pomegranates, oranges (F1)), Vegetables and common Fruits (salads, tomatoes, pears, bananas, grapes, lettuce (F2)), Peppers (yellow, orange and red peppers (F3)), Nuts and Dried fruits (F4), Smoking (F5), and Meals cooked with olive oil and Legumes (F6), which explained 58.27% of the total variance.

The trait anxiety by STAI comprised of 20 items, which was further reduced into three items (Table **S2**), *i.e*., negative thoughts and worries explained by questions 5, 9, 12, 17 and 18 (Negative), positive thoughts and feelings explained by questions 1, 6, 7, 13, 16, and 19 (Positive), and strong emotions comprised of questions 3, 10, 15 and 20 (Emotional), explaining 59.78% of the total variance.

On the other hand, the original brief-COPE by Carver (1997) comprised of 28 questions organised into two-item 14 subscales, including self-distraction, active coping, denial, substance use, use of emotional support, use of instrumental support, behavioural disengagement, venting, positive reframing, planning, humour, acceptance, religion and self-blame. These were further reduced into seven subscales by factor analysis, retaining some subscales or combining some already proposed subscales. In detail, the combination of 1) use of emotional/instrumental support (questions 5, 10, 15, 23, 2) denial/behavioural disengagement (questions 3, 6, 8, 16 and 3) positive reframing/behavioural disengagement (questions 12, 17, 18), the already used subscales of 4) substance use (questions 4, 11, 5) religion (questions 22, 27) and 6) self–blame (questions 13, 26) and a new subscale comprising of questions 24 and 25, included in the subscales of acceptance and planning. These subscales explained 68.21% of the total variance (Table **S3**).

### 2.3 Statistical Analysis

Continuous variables are presented as mean value ± standard deviation, while categorical variables are presented as absolute and relative (%) frequencies. The normality of data was assessed through the Kolmogorov-Smirnov test. Differences were evaluated through the independent t-test and the One-way ANOVA for normal continuous variables and through the Kruskall-Wallis test for non-normal variables, whereas for categorical variables, differences were assessed through the Pearson’s *χ*^2^ Test. The structure of the three questionnaires (dietary/lifestyle, STAI-T and brief-COPE) were studied with (FA) with the principal component analysis method using the varimax rotation for extraction. The Kaiser-Meyer-Olkin (KMO) measure of sampling adequacy and Bartlett’s test of sphericity were computed to determine the fitness of FA [24]. Eigenvalues greater than one and items explaining more than 5% of the variance were retained. Multiple loading of a dimension (>3) or loading lesser than 0.3 was excluded from the FA analysis [25]. For each dimension, the internal consistency reliability was assessed by Cronbach’s alpha coefficient [26].

The association between all the variables in the questionnaires was estimated by multiple regression models. Stepwise multiple regression models were performed, and only the models with the independence of observation (Durbin-Watson~2), linear relationship, homoscedasticity, absence of collinearity issues (variance inflation factors less than 10) and highly influential points, as well as normal distribution were considered. The ones with the best and significant adjusted R^2^ and significant coefficients, are presented for each dependent scale. The statistical analysis was conducted through the IBM SPSS Statistical 24.0 (SPSS. Chicago. IL. USA) and p values ≤0.05 were considered statistically significant in all cases.

## 3 RESULTS

### 3.1 Descriptive Statistics

The socio-demographic characteristics of the 693 participants illustrated in Table **1**, showed that there were at approximately the same frequency males and females, with a mean age of 31.73 years; the majority (71.6%) were between 18 to 34 years of age. Furthermore, most of the participants were single (65.0%), at a superior educational level (64.6%), and unemployed (39.3%).

Females scored significantly higher in the STAI questionnaire than males and middle education, unemployment and retirement/other significantly increased STAI’s total scores, in comparison to all the other educational and working statuses. In addition, the total trait STAI-X-2 scores revealed that one-third of the population (33.5%) understudy had high anxiety and another third (34.4%) moderate anxiety (data not shown), which was also indicated by the populations’ mean score (41.44 ± 7.94).

### 3.2 Association of Socio-demographic Characteristics with the Dietary, Lifestyle Factors

Multiple linear regression was calculated to predict the consumption of each of the dietary factors based on the demographic and socioeconomic characteristics, displayed in Table **2**. Sex and age significantly predicted the weekly consumption of fruit (cherries, watermelon, apricots, tangerines, pomegranate and oranges explained by dietary factor 1) (F(3, 359)=7.895, p<0.0005, adj. R^2^=0.054). In detail, the predicted consumption of fruit is greater in females (by 0.301) than males. Older aged individuals (>35 yrs of age) should increase fruit (by 0.010), salad and common fruit (pear, banana, grapes) consumption and pepper consumption, (F (2, 361) = 18.533, p<0.0005, adj. R^2^=0.088). Living in Sparta significantly predicted higher (by 0.278) pepper weekly consumption (explained by dietary factor 3), (F (2, 334) = 11.857, p=0.010, adj. R^2^=0.027) and consumption (by 0.601) of vegetables cooked in olive oil and legumes (explained by dietary factor 6) [F (1, 359) = 36.097, p<0.0005, adj. R^2^=0.089]. In accordance, family status (being married or cohabitating) predicted being a smoker or higher exposure to smoking, (F (3, 379) = 8.096, p<0.0005, adj. R^2^=0.060).

### 3.3 Association of the Demographic and Socio-economic Characteristics Dietary and Dietary/ Lifestyle Factors with STAI

Multiple linear regression predicted the total scores of STAI, and the scores for each of the STAI factors based on the demographic and socioeconomic characteristics, as well as the scores of the dietary/lifestyle factors, as illustrated in Table **3**. Consumption of lettuce associated with increases in STAI negative and STAI total scores. In contrast, fruit (pears, bananas and grapes) and tomato consumption associated with decreases in negative and total STAI scores and increases in positive STAI.

Use of emotional/instrumental support, denial/behavioural disengagement, substance use and self-blame increased negative, emotional and total STAI scores. On the contrary, positive reframing/humour decreased negative and total STAI scores, but increased STAI positive scores. STAI negative and STAI total was positively associated with being a woman, STAI negative with being younger at age and STAI emotional with living in Kalamata.

A multiple linear regression was calculated in order to predict the use of the brief-COPE factors according to the demographic and socioeconomic characteristics, as well as the lifestyle/dietary factors, and the total and factor scores for STAI, as displayed in Table **4**. The only dietary/lifestyle factor associated with coping was smoking/exposure to smoking that increased as expected substance use, but decreased practice of religion. As far as trait anxiety and its subscales are concerned, negative STAI scores increased use of emotional/instrumental support and denial/behavioural disengagement and decreased use of positive reframing. Furthermore, positive reframing, self-blame and acceptance planning were associated positively with emotional STAI scores. Whereas, positive STAI scores predicted higher emotional/instrumental support, positive reframing/humour and acceptance/planning, but lower denial/behavioural disengagement. Among the sociodemographic covariates, being a woman related to more use of emotional/instrumental support and less substance use and being older is associated with less substance use and more acceptance/planning. Living in Sparta (small town) is related to the use of emotional/instrumental support and positive reframing/humour, and the participants with higher education used more self-blame.

## 4 DISCUSSION

The analysis of dietary/lifestyle patterns under the prism of self-reported trait anxiety (STAI-X-2 inventory) and coping strategies (brief-COPE), was applied in a group of healthy adults, with no known anxiety diagnosis, from the prefectures of Laconia and Messenia in Greece.

Consumption of fruits and tomatoes, is believed to be healthy nutritional choices, associated with lower scores of trait anxiety (both total and negative) and higher scores of positive STAI. It was also of interest that lettuce consumption predicted higher scores of trait anxiety (total and negative). Bioactive ingredients in tomatoes, such as gamma-aminobutyric acid (GABA), esculeoside A, and lycopene, as well as vitamins A, B and C, can restore fatigue, control levels of blood sugar, have anti-oxidants effects and have been shown to have anxiolytic effects [21]. In accordance, phytochemicals found in fruit and vegetables, including vitamins C, E and K, B-group vitamins, carotenoids and phenolic compounds, may play a role in reducing stress levels, in agreement with the current results [22]. Perceived trait anxiety was not associated with changes in fruit and vegetable consumption, suggesting that higher fruit and tomato intake (*via* their constituents), may reduce perceived stress and not that anxiety had an effect on the dietary patterns of the participants. This is in agreement with other research that has associated consumption of fruit and vegetable with a reduction in anxiety levels [22]. On the contrary, the lettuce consumption paradox may be attributed to increased copper ions usually found in industrialized cultivations of lettuce; these have been associated in a previous study with increased anxiety symptoms [23, 24].

On the other hand, only smoking/exposure to smoking is associated with the coping strategies, increasing substance use but decreasing religious practices. Religion practices are often characterized by structure and rules that usually restrain individuals from substance use and hence smoking/exposure to smoking [25]. However, it is evident that the population’s, understudy coping strategies were not affected by the dietary/lifestyle patterns, as observed in previous studies [26].

As far as trait anxiety and coping are concerned, the combination of maladaptive coping strategies, like denial/behavioural disengagement, substance use and self-blame, is associated in our study group with higher scoring in total, as well as negative and emotional trait anxiety. These are often referred to in the literature as avoidance coping and have been related to higher anxiety levels. Whereas more adaptive strategies, such as positive reframing/humour and acceptance planning, often described as positive coping, are associated with better positive STAI scores and lower negative and total scores. In addition, seeking emotional/instrumental support was associated with both positive STAI, as well as negative STAI, reflecting each dual role in problem-focused solving, which is not active but a step towards solving.

Some coping styles may contribute to anxiety vulnerability and may also be strong predictors of anxiety symptomatology, with greater emotion-oriented coping being reported by individuals with higher anxiety [27]. Instrumental or emotional support in some studies is categorised as a maladaptive, emotional-focused strategy and, in others, is reported as a positive, adaptive approach to anxiety coping. Furthermore, emotion-focused coping is usually predictive of higher anxiety and functional impairment in clinical and non-clinical contexts [27-30]. This controversy might be partially explained by the notion that searching support, although in itself may help manage and reduce stress, might at the same time trigger or allow the maintenance of maladaptive strategies, such as self-blame and denial [27, 28]. On the contrary, problem-solving and changing perspective appears to be a valid approach to anxiety moderation and psychopathology in general and has been associated with better mental health outcomes [29].

Fruit consumption associated with women did not counteract their increased anxiety levels in comparison to men, possibly due to the higher stress vulnerability related to women. In particular, emotion-focused coping strategies are considered to be a more female role-orientated strategy, as previously demonstrated by higher expression of positive feelings, greater responsiveness to emotional stimuli and higher vulnerability to anxiety [31]. Healthier eating habits are often associated with women more than men, but the amount and frequency of healthier choices might not be able to control anxiety responses, in more vulnerable individuals and could reflect the need for more profound changes and richer in macronutrients and oligo-elements, dietary habits.

Older participants exhibited higher consumption of fruits and salads, better STAI negative scores, and use of acceptance planning, characteristics that all individually contribute to decreased anxiety. Although healthier eating habits seem to establish over the whole lifespan, they are more frequently adopted in older individuals than younger adults who tend to choose more high-fat and high-sugar products and follow a more Westernized diet [32]. Younger age, may also explain the lack of anxiety resilience and use of an avoidance strategy like substance use, whereas acceptance and planning are more of an active problem-solving strategy that is found in the more experienced older in age individuals [33], whereas coping strategies, for the younger are usually dependent more on the stressors [34].

Urban living (Sparta residents), is associated with higher consumption of peppers, legumes and vegetables cooked in olive oil, smaller emotional STAI and use of emotional/instrumental support and positive reframing/humour. Living in a small city in Greece increases the likelihood of higher adherence to the Mediterranean style eating [35], may impose a lower emotional burden, than that reported by residents of a large city, possibly reflecting the benefits of a smaller and greener urban environment [36] and provides a closer social environment, more easily accessible for support, without the deprivation of education, employment and access to specialised care offered by city living [37].

Some limitations of the present study should be considered. Although the present findings according to the sociodemographic characteristics are in agreement with previous studies, they should be interpreted with caution due to the fact that the majority of the studied population that accepted to participate and fill up the questionnaires were younger in age, mostly at a superior educational level, single and unemployed, which is not the case for the general population of Greece. Therefore, further studies in a more balanced population might confirm the relationships observed and may elucidate the association of anxiety with sociodemographic status in Greece. For these reasons, results cannot be generalized. Also, self-reported questionnaires are limited by biases such as question interpretation, introspective ability and honesty. Since the participants were not followed for a period of time in the present study, the causal relationship between dietary/lifestyle patterns and anxiety should be interpreted with caution. Further exploration of the population under study could elucidate the existence of a true relationship. Furthermore, anamnestic features, like the presence of an anxiety disorder or other psychiatric disorder was not evaluated clinically, and to our knowledge, none was present, but the anxiety scores should be interpreted with caution. Only 1/3 of the participants, though, scored high (≥45) for trait anxiety. In addition, the factor analysis of brief-COPE excluded factors that are described in the literature to contribute to active problem-solving strategies and emotional expression. These strategies are often proposed to beneficially contribute to anxiety reduction and resilience and can inverse the effects of anxiety following stressful events in everyday life [38]. Therefore, their inclusion might have provided more insightful ways of nutritional and anxiety-orientated coping.

In the current study, women, younger (≤34 years of age) individuals, living in larger cities, married or cohabitating, with a superior educational status, might be at a higher risk (approximately 1 fold increase in trait anxiety scores) for developing subclinical anxiety and maladaptive coping practices and thus, would benefit more from future interventions.

## CONCLUSION

The discussion of the results of this study provides meaningful information to support the necessity of developing interventions that not only enhance and support a healthier lifestyle in terms of dietary patterns, quality sleeping, and physical activity, but also attempt to enhance positive and adaptive coping strategies. Strategies, such as active problem solving, positive reframing, humour, acceptance and planning, together with a healthier lifestyle and dietary habits, might improve anxiety and coping with anxiety-related events, but it might also limit the use of maladaptive coping strategies, contributing therefore to an overall healthier body and mind. Maladaptive strategies, like denial and behavioural disengagement, substance use and self-blame may limit the positive effects of other daily habits and, in some cases, counteract anxiety coping.

## Figures and Tables

**Table 1:** Socio-demographic characteristics of the adults living in the prefectures of laconia and messenia and mean STAI summary score according to these characteristics.

-	-	**N(%)** **(Total 693)**		**Total STAI Score^a^** 41.44 ± 7.941^a^
**Sex**	Male	345(49.8)		40.63 ± 7.93
	Female	348(50.2)		42.75 ± 7.97
p value	-	-	-	0.001
**Age (yrs)**	18-34	446(71.6)		42.33±7.88
*31.73 ±13.93^a^*	35-44	72(10.3)		39.80 ±6.70
-	45-54	46(6.6)		43.22 ± 8.31
-	55-64	43(6.1)		39.02 ±9.30
-	65+	16(2.3)		39.43 ± 11.49
p value	-	-	-	≤0.043
**Family status**	Single	443(65.0)		42.23 ±8.15
	Married/Cohabitation	219(32.1)		40.52 ± 8.03
	Separated/Divorced/Widowed	20(2.9)		41.11 ± 8.09
p value	-	-	-	NS
**Place of Residence**	Big City (Kalamata)	367(59.1)		41.57 ± 8.15
	Smaller city (Sparta)	254(40.9)		41.93 ± 8.03
p value	-			NS
**Education**	Primary	177(26.1)		40.43 ± 7.80
	Superior	438(64.6)		42.45 ± 8.03
	Post-graduate	63(9.3)		40.29 ± 8.15
p value	-	-	-	0.037
**Work Status**	Unemployed	251(39.3)		42.99 ± 7.35
	Private/Civil servant	207(32.4)		40.85 ± 7.85
	Self-employed	55(8.6)		40.41 ± 9.25
	Farmer	11(1.7)		39.87 ± 6.87
	Retired/Other	115(18.0)		41.63 ± 9.13
p value	-	-	-	0.031

*^a^Mean ± standard deviation*.

Differences tested by Kruskal-Wallis and Mann-Whitney U, were appropriate.

**Table 2:** Multiple linear regression models for the dietary/lifestyle factors according to demographic characteristics and socioeconomic status.

**Dietary/lifestyle**	**β (SE)^1^**	-	-	-	-
	**F1**	**F2**	**F3**	**F5**	**F6**
**Demographic**	-	-	-	-	-
Sex	0.301(0.107)**	-	-	-	-
Age	0.010(0.004)**	0.015 (0.003)***	0.009(0.004)*	-	-
	-	-	-	-	-
**Socioeconomic**	-	-	-	-	-
Place of Residence	-	-	0.326(0.117)**	-	-0.601(0.100)***
Family Status	-	-	-	-	-
Single	-	-	-	-0.425(106)***	-
Married/cohabitate	-	-	-	0.432(109)***	-
	-	-	-	-	-
**Adjusted R^2^**	0.054***	0.088***	0.027**	0.06*** 0.06***	0.089***

^1^β (SE):non standardized regression coefficient (coefficients standard error).

p values:*p≤0.05, **p≤0.01, ***p≤0.0001.

F1:Fruits, F2: Vegetables and common Fruits, F3:Peppers, F4: Nuts and Dried fruits, F5: Smoking habits/exposure, F6: Mediterranean meals.

**Table 3:** Multiple linear regression models for the STAI factors and total score according to the demographic characteristics, socioeconomic status, lifestyle dietary variables and brief-COPE factors.

-	**β (SE)^1^**	-	-	-
-	**STAI Negative**	**STAI Positive**	**STAI Emotional**	**STAI Total**
**Demographic**	-	-	-	-
Sex	0.479 (0.154)***	-	0.387 (0.132)**	1.816 (0.551)**
Age	-0.012 (0.006)**	-	-	-
**Socioeconomic**	-	-	-	-
Place of Residence	-	-	-0.516(0.135)***	-
Work Status	-	-	-	-
Unemployed	-	-	-	-
**Dietary/lifestyle**	-	-	-	-
Weekly Fruits (pear, banana, grapes)	-0.088 (0.03)**	0.088(0.04)*	-	-0.457(0.129)***
Weekly tomato	-0.09 (0.039)*	0.159(0.04)***	-	-0.432(0.144)**
Weekly lettuce	0.087 (0.042)*	-	-	0.340(0.155)*
Weekly Salad	-	-	-	-
**Brief COPE**	-	-	-	-
use of Emotional/ Instrumental Support	0.327 (0.247)***	-	0.138 (0.062)*	0.643 (0.271)*
Denial/Behavioural Disengagement	0.983 (0.07)***	-0.657 (0.08)***	0.388 (0.059)***	3.728 (0.271)***
Positive Reframing/ Humour	-0.397 (0.07)***	0.466 (0.08)***	-	-1.880(0.260)***
Substance Use	0.27 (0.07)***	-0.245 (0.08)**	0.162 (0.059)**	0.999(0.264)***
Self-Blame	0.446 (0.07)***	-0.373 (0.08)***	0.419 (0.059)***	1.674 (0.551)***
Acceptance/Planning	-	0.323 (0.08)***	-	-
**Adjusted R^2^**	0.374***	0.246***	0.178***	0.390***

^1^β (SE):non standardized regression coefficient (coefficients standard error).

p values:*p≤0.05, **p≤0.01, ***p≤0.0001.

**Table 4:** Multiple linear regression models for the brief-COPE factors according to demographic characteristics, socioeconomic status, lifestyle dietary variables and STAI factors.

-	**β (SE)^1^**	-	-	-	-	-	-
-	**use of Emotional/** **Instrumental Support**	**Denial/Behavioural** **Disengagement**	**Positive Reframing/** **Humour**	**Substance** **use**	**Religion**	**Self-Blame**	**Acceptance/Planning**
**Demographic**	-	-	-	-	-	-	-
*Sex*	0.280 (0.089)**	-	-	-0.374 (0.0.084)**	-	-	-
*Age*	-	-	-	-0.01(0.003)***	-	-	0.022(0.003)***
	-	-	-	-	-	-	-
**Socioeconomic**	-	-	-	-	-	-	-
Place of Residence	0.430(0.089)***	-	0.327(0.088)***	-	-	-	-
Education	-	-	-	-	-	-	-
Lower	-	-	-	-	-	-0.217(0.091)*	-
Medium	-	-	-	-	-	0.172 (0.083)*	-
	-	-	-	-	-	-	-
**Dietary/Lifestyle**	-	-	-	-	-	-	-
Smoking/exposure to smoking	-	-	-	0.315(0.079)***	-0.2 (0.078)***	-	-
	-	-	-	-	-	-	-
**Trait anxiety**	-	-	-	-	-	-	-
Positive	0.087 (0.021)***	-0.063 (0.018)**	0.105(0.022)***	-	-	-	0.079(0.02)***
Negative	0.116(0.087)***	0.154 (0.019)***	-0.101(0.027)***	-	-	-	-
Emotional	-	-	0.112(0.036)***	-	-	0.142 (0.03)***	0.078(0.028)***
	-	-	-	-	-	-	-
**Adjusted R^2^**	0.148***	0.218***	0.112***	0.122***	0.008***	0.102*** 0.104*** education	0.124***

^1^β (SE):non standardized regression coefficient (coefficients standard error).

p values:*p≤0.05, **p≤0.01, ***p≤0.0001.

## Data Availability

The data supporting the finding of the article is available in the Zenodo Repository at zenodo.org, reference number https://doi.org/10.5281/zenodo.5130583.
