# Supplementary material for: Anxiety Status and Coping Strategies in Association with Sociodemographic Factors, Dietary and Lifestyle Habits in Greece
Source: Clin Pract Epidemiol Ment Health. 2021 Oct 15;17:152–60. doi: 10.2174/1745017902117010152 (PMC8595982; doi:10.2174/1745017902117010152)
Supplement: Supplementary file 1 — Supplementary material is available on the publishers web site along with the published article. [file CPEMH-17-152_SD1.pdf]

1745-0179/21 *Send Orders for Reprints to reprints@benthamscience.net*

Clinical Practice & Epidemiology in Mental Health
Supplementary Material
Content list available at: https://clinical-practice-and-epidemiology-in-mental-health.com

Anxiety Status and Coping Strategies in Association with Sociodemographic Factors, Dietary and Lifestyle Habits in Greece

Maria Batsikoura[1], Sofia Zyga[2], Fotini Tzavella[3], Athanasios Sachlas[4] and Andrea Paola Rojas Gil[1,*]

[1]*Department of Nursing Laboratory of Biology and Biochemistry, Faculty of Health Sciences,, University of Peloponnese, Tripoli, Greece*
[2]*Department of Nursing, Laboratory of Basic Nursing, Faculty of Health Sciences, University of Peloponnese, Tripoli, Greece*
[3]*Department of Nursing, Laboratory of Integrated Health Care, Faculty of Health Sciences, University of Peloponnese, Tripoli, Greece*
[4]*Department of Statistics and Insurance Science, University of Piraeus, Athens, Greece*

Table S1. PCA for the dietary and lifestyle questionnaire.

Weekly consumption of	F1	F2	F3	F4	F5	F6
Cherries	0.780	-	-	-	-	-
Watermelon	0.713	-	-	-	-	-
Apricots	0.672	-	-	-	-	-
Tangerines	0.643	-	-	-	-	-
Pomegranate	0.565	-	-	-	-	-
Oranges	0.564	-	-	-	-	-
Salad	-	0.820	-	-	-	-
Tomato	-	0.669	-	-	-	-
Fruits (pear, bananas, grapes)	-	0.642	-	-	-	-
Lettuce	-	0.623	-	-	-	-
Yellow/Orange pepper	-	-	0.808	-	-	-
Red pepper	-	-	0.798	-	-	-
Pepper	-	-	0.696	-	-	-
Nuts	-	-	-	0.814	-	-
Dried Fruits	-	-	-	0.758	-	-
Cigarettes	-	-	-	-	0.848	-
Smoker/exposed to smoke	-	-	-	-	0.828	-
Vegetables cooked in olive oil	-	-	-	-	-	0.808
Legumes	-	-	-	-	-	0.789
**Cronbach's alpha total 0.836**	0.762	0.691	0.768	0.614	0.611	0.605

F1:Fruits, F2: Vegetables and common Fruits, F3:Peppers, F4: Nuts and Dried fruits, F5: Smoking habits/exposure, F6: Mediterranean meals.

Table S2. PCA for trait anxiety (STAI-X-2) scales.

-	Negative	Positive	Emotional
I take disappointments so keenly that I can't put them out of my mind (Q18)	0.665	-	-
I lack self-confidence (Q12)	0.635	-	-
I feel that difficulties are piling up so that I cannot overcome them (Q8)	0.627	-	-

DOI: 10.2174/1745017902117010152, 2021, *17*, i-ii

(Table S2) contd.....

Some unimportant thoughts runs through my mind and bothers me (Q17)	0.620	-	-
I worry too much over something that really doesn't matter (Q9)	0.609	-	-
I am losing out on things because I can't make up my mind soon enough (Q5)	0.561	-	-
I feel secure (Q13)	-	0.696	-
I feel pleasant (Q1)	-	0.686	-
I am content (Q16)	-	0.684	-
I am "calm, cool, and collected" (Q7)	-	0.643	-
I feel rested (Q6)	-	0.628	-
I am a steady person (Q19)	-	0.534	-
I am happy (Q10)	-	-	0.743
I feel blue (Q15)	-	-	0.651
I feel like crying (Q3)	-	-	0.609
I become tense and upset when I think about my present concerns (Q20)	-	-	0.476
**Cronbach's alpha: total 0.608**	0.758	0.757	0.688

Table S3. PCA for the Brief COPE.

-	Use of Emotional/ Instrumental Support	Denial/ Behavioural Disengagement	Positive Reframing/ Humour	Substance use	Religion	Self-Blame	Acceptance /Planning
Q10	0.832	-	-	-	-	-	-
Q15	0.823	-	-	-	-	-	-
Q5	0.818	-	-	-	-	-	-
Q23	0.811	-	-	-	-	-	-
Q8	-	0.766	-	-	-	-	-
Q16	-	0.734	-	-	-	-	-
Q6	-	0.688	-	-	-	-	-
Q3	-	0.562	-	-	-	-	-
Q17	-	-	0.789	-	-	-	-
Q12	-	-	0.736	-	-	-	-
Q18	-	-	0.731	-	-	-	-
Q4	-	-	-	0.913	-	-	-
Q11	-	-	-	0.885	-	-	-
Q27	-	-	-	-	0.856	-	-
Q22	-	-	-	-	0.850	-	-
Q13	-	-	-	-	-	0.800	-
Q26	-	-	-	-	-	0.772	-
Q24	-	-	-	-	-	-	0.834
Q25	-	-	-	-	-	-	0.616
**Cronbach's alpha: Total 0.738**	0.853	0.660	0.671	0.814	0.683	0.544	0.494

© 2021 Batsikoura *et al*.

This is an open access article distributed under the terms of the Creative Commons Attribution 4.0 International Public License (CC-BY 4.0), a copy of which is available at: (https://creativecommons.org/licenses/by/4.0/legalcode). This license permits unrestricted use, distribution, and reproduction in any medium, provided the original author and source are credited.
